# Malaria prevalence and associated population and ecological risk factors among women and children under 5 years in Rwanda

**DOI:** 10.1016/j.heliyon.2024.e34574

**Published:** 2024-07-14

**Authors:** Guillaume Rudasingwa, Sung-il Cho

**Affiliations:** a Department of Public Health Sciences, Graduate School of Public Health, Seoul National University, Seoul, 08826, South Korea; b Institute of Health and Environment, Seoul National University, Seoul, 08826, South Korea

**Keywords:** Malaria, Irrigation, Livestock, Africa, Vector-borne diseases, Epidemiology

## Abstract

**Background:**

Malaria continues to pose a substantial public health concern in Rwanda, despite substantial progress in recent years. Little is known about effect of ecological factors and their interaction in malaria transmission. Understanding the prevalence and identifying risk factors, both population-based and ecological such as zooprophylaxis and irrigation are crucial for targeted intervention strategies.

**Methods:**

This study analyzed the 6th iteration of the Demographic and Health Survey conducted in Rwanda between 2019 and 2020. The study employed a nationally representative sample, utilizing rapid diagnostic tests and blood smear microscopy to determine malaria prevalence among women and under 5 years old children. Logistic regression analysis was used in R version 4.3.1 to evaluate population and ecological risk factors that are associated with malaria prevalence. Additionally, interactive effects of ecological factors on malaria were evaluated.

**Results:**

The analysis revealed a notable malaria prevalence in Rwanda, emphasizing the continued significance of malaria control efforts. Approximately 1.79 % of the population tested positive for malaria. Proximity to irrigation sites and lowland were identified as significant risk factors to malaria with adjusted odds ratio (AOR) 1.47(1.00–2.15) and AOR 5.44(4.01–8.61) respectively however cattle ownership exhibited a protective effect AOR 0.41(0.23–0.72). Interactive effects of livestock and irrigation on malaria prevalence were revealed. Additionally, population-based risk factors, including age, household wealth, utilization of Insecticide Treated Nets, were associated with varying malaria risks.

**Conclusion:**

This study underscores the persistent challenge of malaria in Rwanda and the importance of tailored intervention strategies. To effectively combat malaria, efforts must consider the interplay of ecological factors, such as high cattle density, and demographic factors, targeting high-risk populations, especially those living in proximity to lowlands and irrigation areas. These findings provide critical insights for advancing malaria elimination efforts in Rwanda and serve as a basis for comprehensive public health planning and action.

## 1 Introduction

Malaria remains a significant public health concern in many African countries, including Rwanda [1]. Understanding malaria population and ecological risk factors in Rwanda is critical for furthering malaria elimination efforts in Africa. To manage the transmission as well as lessening the impact of malaria, it is essential to identify contributing risk factors of malaria infection. These factors can be utilized to identify individuals or communities with a higher likelihood of contracting the disease and, as a result, can benefit from targeted programmatic interventions [2].

Despite substantial efforts to control and eliminate malaria in Rwanda, it remains a significant factor contributing to illness and death, particularly among vulnerable populations such as pregnant women and children under the age of five [3].

Understanding the factors contributing to malaria prevalence is essential for designing effective control and prevention strategies [3]. Malaria transmission is a complex interplay of various factors, including human behavior, environmental conditions, and ecological factors [4]. In Rwanda, as in many sub-Saharan African countries, malaria transmission is influenced by a combination of socio-demographic, environmental, and ecological factors [5,6].

Malaria parasite infection is determined by a combination of factors related to mosquitoes and humans. Moreover, ecological factors like land cover, rainfall, altitude, and temperature significantly affect mosquitoes’ breeding, which, in turn, affects malaria transmission risks. Regions with higher levels of rainfall and elevated temperatures are anticipated to exhibit higher malaria prevalence, as these conditions promote the breeding of various anopheline species and facilitate the reproduction of parasites within the mosquitoes [7,8]. Agriculture and urban development can also impact malaria transmission. Extensively cultivated areas provide more favorable habitats for the primary vectors, which thrive in non-forest environments and prefer sunlit conditions. On the other hand, urbanized regions generally have reduced breeding grounds for vectors; however, in certain cases, poor sanitation conditions in urban areas may encourage vector breeding [9,10].

Presence of animals like cattle within and close to human dwellings can potentially redirect mosquitoes from biting humans, thus reducing the transmission of the parasite however the protective effect of cattle remains debatable [11]. The idea of employing different host species to draw malaria vectors away from humans, a concept referred to as zooprophylaxis, has been suggested as a potential environmental approach to mitigate malaria transmission [12,13]. Nonetheless, when the animal population grows, the increased accessibility of blood meals might enhance mosquito survival, thus offsetting the effects of diverting feeds [14].

As we strive to combat vector-borne diseases like malaria, it is imperative to recognize the intricate interplay between various ecological factors and disease transmission dynamics. Therefore, there is a crucial need for more detailed studies that delve into mosquito behavior, the ecological impact of irrigation, the role of livestock in local ecosystems, and their intricate interactions.

As little is known about the effect of ecological factors and their interaction on malaria transmission in Rwanda, this study aims to examine malaria prevalence in Rwanda and the association between malaria with a range of population and ecological factors. By delving into the multifaceted nature of malaria transmission, this research aims at providing meaningful understandings that can guide interventions grounded in empirical evidence as well as policies with the goal of reducing the malaria burden in Rwanda.

## 2 Methods

### 2.1 The 2019–2020 Demographic and Health Survey in Rwanda

The DHS conducted in 2019–2020 marked the 6th iteration of the Demographic and Health Survey(DHS) series carried out in Rwanda which is a sub-Saharan African country with approximately 13 million of population. Data collection for this survey spanned from November 9, 2019, to July 20, 2020. However, it's worth noting that the data collection faced a disruption lasting over two months due to the COVID-19 pandemic lockdown from march to June in 2020 in Rwanda. Centers for Disease Control and Prevention (CDC), United States Agency for International Development (USAID) as well as United Nations (UN) Women funded this survey [15]. 26 households were chosen from each sampling location, resulting in a combined sample size of 13,000 households [15].

### 2.2 Ethic statement

Institutional Review Board of Seoul National University (IRB No. E2403/002–018) approved this study and the reporting of study findings followed the STROBE guidelines [16].

### 2.3 Survey field staff training

The primary training commenced on September 30th and concluded on November 1, 2019. Around 160 individuals from various regions across Rwanda participated in the training. Among the participants, 85 were assigned the roles of interviewing participants and leading survey teams, while 51 were appointed as health professionals [15].

### 2.4 Field work

The data collection process was executed by 17 field teams formed after training, each team was equipped a vehicle and a designated driver. To ensure timely and secure transfer of blood smears and dried blood spots (DBS).

Specimens were transported to the National Institute of Statistics of Rwanda (NISR) office by supervisors. These team leaders also played a crucial role in coordinating and supervising fieldwork activities. In the field, venous blood specimens were processed within field laboratories established at referral hospitals at the district level. Mobile freezers were used to store serum aliquots at −20 °C prior to their transfer to regional laboratories in order to preserve the integrity of the samples during transport and, afterwards, to the National Reference Laboratory (NRL). Throughout data collection phase, practical support was provided by Inner City Fund (ICF) and the CDC [15].

### 2.5 Data preprocessing

Data preprocessing commenced almost immediately after the initiation of fieldwork. Upon completion of data collection in every cluster, all digital data files were sent to the NISR central office in the City of Kigali. These data files underwent thorough registration and examination to identify any inconsistencies, gaps, or unusual data points. Any discrepancies and errors detected were promptly communicated to the field teams for resolution. Further data refinement, termed secondary editing, was conducted at the central office. This process consisted of addressing discrepancies and encoding answers to questions that allow for open-ended responses. Coordination of this exercise at the central office was overseen by the NISR data processor. To ensure data accuracy, a comparison was made between the paper-based biomarker questionnaires and the digital data files to identify and rectify disparities in the process of inputting data and editing using the Census and Survey Processing System (CSPro) [15].

### 2.6 Sample design

**Fig. 1:**
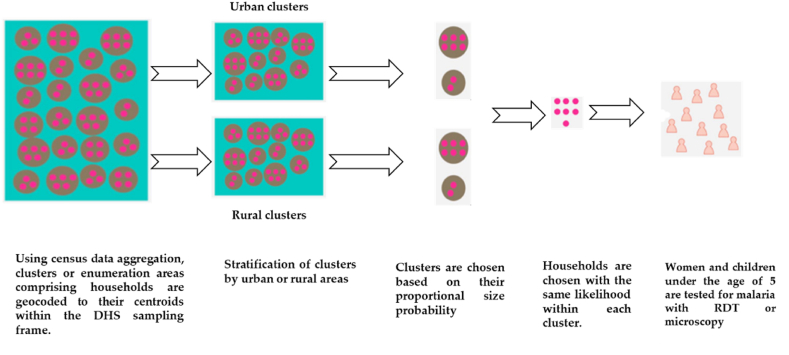
Framework of Rwanda DHS design and sampling.

The survey sampling frame was based on the population census conducted in 2012. This frame comprises a comprehensive list of enumeration areas covering Rwanda as a whole [15]. The survey design for the 2019-20 Rwanda DHS was two-staged, with the aim of facilitating estimations of key indicators at various levels, including national, urban, rural, provincial (five provinces), and district levels for a select set of indicators. The initial stage involved the selection of sample points, referred to as clusters, comprising enumeration areas(EAs) demarcated during the population census. The selection involved a total of 500 clusters, with 112 and 388 regions from urban and rural settlements respectively [15](Fig. 1).

In the second phase of sampling, households were systematically selected. A comprehensive listing of households took place in all the designated EAs between June and August 2019. The survey then randomly picked households from these lists for inclusion. Specifically, from every sample point, 26 households were picked making up to 13,000 households in survey sample [15].

### 2.7 Malaria testing and eligibility

In the 2019-20 Rwanda DHS, malaria diagnostic assessments, which included both a rapid diagnostic test (RDT) using SD Bioline Malaria Ag P.F/Pan test and blood smear tests involving both thin and thick blood samples, were administered to qualified children and women. For the RDT, a small sample of blood was collected by puncturing the tip of the finger. These RDT outcomes were instrumental in diagnosing malaria and guiding children treatment found to have malaria parasites during the survey. Women who tested positive were treated. Eligible women, parents, or responsible adults were given detailed information through an informed consent form [15].

Furthermore, blood samples from individuals who consented to undergo malaria testing were collected among women between 15 and 49 years and children under the age of 5. These samples were meticulously dried, securely for microscopic examination for malaria infection identification [15].

### 2.8 Variables

#### 2.8.1 Outcome variable

In this study, the outcome variable was the reported status of malaria infection among children and women. The response was categorized as negative or positive.

#### 2.8.2 Independent variables

The selection of independent variables was based on the review of literature [1,17] and the availability of relevant data in the Rwanda DHS 2019–2020 dataset. A total of 12 sociodemographic variables, and 3 ecological variables were included as independent variables.

The study considered various sociodemographic variables, such as the age of the participants (15–25, 26–35, 36–49), educational attainment (No education, primary, higher), household wealth category (very low, low, middle, higher, highest), type of residence (rural, urban), province of residence (Kigali city, Western, Eastern, Northern and Southern provinces), Having insurance (yes or no), type of insurance (Community health insurance or private insurance). Additionally, lifestyle-related factors were taken into account, including use of Insecticide-Treated Nets (ITN) classified as yes or no. Ecological factors such as Irrigation Non-irrigated and Irrigated), heads of livestock especially Cattle per square kilometer (<5, >5), Season (long rainy season: between the month of march and may, and short rainy season: between the month of September and November, and long dry season: starting in June and ending by August as well as and the short dry season: from December up to February) and Elevation above the sea level (<1700 m, <1700 m).

### 2.9 Data analysis

#### 2.9.1 Descriptive statistics

Variables were initially assessed for normality though Q-Q plots. Descriptive statistics were employed, calculating percentages, to determine the frequency of malaria cases based on general characteristics. The proportion of malaria cases was estimated using Rwanda DHS sample weights, adjusting for the cluster survey design. Using Chi-square test, the significance between and each variable and malaria status were assessed.

##### 2.9.1.1 Association between malaria and population and ecological factors

A logistic regression model was employed to investigate the factors influencing malaria prevalence in Rwanda. The association between malaria prevalence and various factors such as ITN usage, age, sex, education, socioeconomic factors, ownership of livestock, irrigation, elevation and residence place was assessed. Significance was considered at a threshold of P < 0.05, and the results are expressed as crude odds ratios (CORs) or adjusted odds ratios (AORs) with corresponding 95 % confidence intervals (CIs). Confidence intervals play a crucial role in understanding the precision and reliability of estimates, such as odds ratios or prevalence rates.

R statistical software were used and the survey package in R that offers various functions and methods for analyzing survey data, taking into account survey design encompassing elements such as clustering, weighting and clustering. svyby(.) and svyglm(.) functions were used for calculating descriptive statistics for survey data and to fit a logistic regression model to the survey data, considering the survey design features respectively.

## 3 Results

### 3.1 Malaria prevalence

Out of 10443 participants (1755 boys and 1756 girls under the age of 5 and 6932 women) who were tested for malaria, 187 (1.79 %) were tested positive (Table 1). The prevalence of malaria showed to high among participants from lowest and low income level, low education level, people not sleeping under insecticide treated nets, people living in lowlands, uninsured as well as people living near irrigation areas. Living close to heads of livestock showed a protective effect to malaria prevalence (Table 1).

**Table 1:** Social-demographic features of the study participants categorized by malaria status.

Variables	Total, N = 10443	Negative, N = 10255	Positive, N = 187 (1.79)	a*(P)*
Sex				<0.001*
Boys	1755	1712	43 (2.45)	
Girls	1756	1698	57 (3.24)	
Women	6932	6845	87 (1.25)	
Age group				<0.001*
<1	416	408	8 (1.92)	
1	746	729	17 (2.27)	
2	792	775	17 (2.14)	
3	776	752	24 (3.09)	
4	781	747	34 (4.35)	
15–25	2910	2863	47 (1.61)	
26–35	2005	1985	20 (0.99)	
36–49	2017	1997	20 (0.99)	
Location				0.065
Urban	1779	1758	21 (1.18)	
Rural	8663	8497	165 (1.90)	
Wealth category				<0.001*
Very low	2142	2085	57 (2.66)	
Low	2198	2144	54 (2.45)	
Middle	2113	2071	42 (1.98)	
Higher	2102	2082	20 (0.95)	
Highest	1887	1873	14 (0.74)	
Level of education				<0.001*
No education	4167	4058	109 (2.61)	
Primary & secondary	4255	4201	54 (1.26)	
University	2018	1996	22 (1.09)	
Provinces				0.234
Kigali	1374	1350	25 (1.81)	
South	2217	2174	44 (1.98)	
West	2392	2335	56 (2.34)	
North	1581	1557	23 (1.45)	
East	2878	2839	39 (1.35)	
ITN				<0.05*
Yes	7253	7147	106 (1.46)	
No	3188	3108	80 (2.50)	
Insurance				<0.001*
Yes	8269	8152	117 (1.41)	
No	2173	2103	70 (3.22)	
Elevation				<0.001*
<1700 m asl	6423	6255	168 (1.60))	
>1700 m asl	4019	4000	18 (0.17)	
Irrigation				0.08
Irrigated	3410	3342	78 (2.00)	
Non-irrigated	7032	6913	109 (1.69)	
Livestock (Cattle) (heads/sqm)				<0.001*
<5	606	578	28 (4.62)	
>5	9836	9677	159 (1.61)	
Season				0.142
Long rainy season	1338	1311	27 (2.01)	
Short rainy season	1099	1082	17 (1.54)	
Long dry season	3157	3115	42 (1.33)	
Short dry season	4848	4747	101 (2.08)	

a *(P):* Chi-squared P value.

**Fig. 2:**
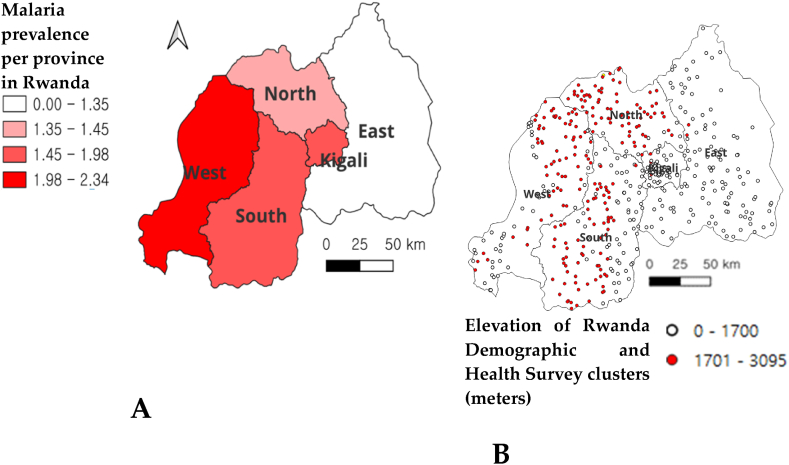
Spatial malaria prevalence rate by province confirmed by the rapid test **(A)** and elevation in meters of Rwanda DHS clusters (**B**).

Malaria had a significant presence in the Western and Southern Provinces, indicating a high prevalence in these regions with 2.34 and 1.98 % respectively. Eastern province as well as Northern province exhibited a low prevalence rate of malaria with 1.35 and 1.45 % respectively (Fig. 2).

### 3.2 The association between malaria and population and ecological factors in Rwanda

Having no formal education and Non-adherence to the Insecticide-Treated Nets(ITN) usage was associated with the malaria prevalence with 2.09, (1.47–2.98) and 1.72, (1.21–2.45) respectively. In addition, being uninsured was positively linked to malaria prevalence 1.96, (1.34–2.86). Household members living at < 1700 m asl as well as living at proximity of irrigation areas showed an association with a significant prevalence of malaria infection with 5.33, (3.21–8.86) and 1.47, (1.00–2.15) respectively. Province of residence, type of season and type of residence were not statistically linked to the prevalence of malaria (Table 2).

**Table 2:** Logistic regression analysis of factors linked with malaria prevalence.

Variables	Crude OR(95 % CI)	Adjusted OR^a^ (95 % CI)
**Sex**		
**Boys**	1	1
**Girls**	1.33(0.85–2.07)	1.35(0.87–2.10)
**Women**	0.50(0.34–0.72)	**0.51(0.35**–**0.77)**
**Age group**		
< **1**	1	1
**1**	1.18(0.37–2.09)	1.17(0.35–2.07)
**2**	1.11(0.47–1.87)	1.10(0.45–1.82)
**3**	1.62(0.76–2.58)	1.70(0.74–2.63)
**4**	2.32(1.05–3.66)	**1.99(1.02**–**3.58)**
**15**–**25**	0.83(0.48–1.27)	0.84(0.44–1.34)
**26**–**35**	0.51(0.24–0.89)	**0.52(0.23**–**0.90)**
**36**–**49**	0.51(0.21–0.86)	**0.46(0.23**–**0.93)**
**Location**		
**Urban**	1	1
**Rural**	1.62(0.96–2.96)	1.37(0.78–2.41)
**Wealth category**		
**Very low**	1.34(0.85–2.11)	1.24(0.79–1.97)
**Low**	1.24(0.76–2.01)	1.19(0.74–1.93)
**Middle**	1	1
**Higher**	0.47(0.24–0.89)	**0.47(0.25**–**0.91)**
**Highest**	0.37(0.17–0.80)	**0.39(0.18**–**0.85)**
**Education level**		
**No education**	2.08(1.46–2.97)	**2.09(1.47**–**2.98)**
**Primary & secondary**	1	1
**Higher**	0.85(0.51–1.44)	0.90(0.53–1.53)
**Elevation**		
< **1700m asl**	5.96(3.03–8.30)	**5.44(4.01**–**8.61)**
> **1700m asl**	1	1
**Provinces**		
**Kigali**	1	1
**South**	1.11(0.58–2.13)	0.94(0.48–1.82)
**West**	1.34(0.69–2.60)	1.11(0.58–2.13)
**North**	0.83(0.39–1.75)	0.69(0.32–1.46)
**East**	0.69(0.40–1.11)	0.63(0.32–1.23)
**ITN**		
**Yes**	1	1
**No**	1.73(1.24–2.48)	**1.61(1.19**–**2.28)**
**Insurance**		
**Yes**	1	1
**No**	2.28(1.57–3.30)	**1.96(1.34**–**2.86)**
**Irrigation**		
**Irrigated**	1.18(0.82–1.69)	**1.47(1.00**–**2.15)**
**Non-irrigated**	1	1
**Livestock (Cattle) (heads/sqm)**		
< **5**	1	1
> **5**	0.35(0.20–0.60)	**0.41(0.23**–**0.72)**
**Season**		
**Long rainy season**	1	1
**Short rainy season**	0.76(0.40–1.51)	0.71(0.37–1.41)
**Long dry season**	0.65(0.36–1.21)	0.66(0.35–1.23)
**Short dry season**	1.03(0.62–1.77)	0.96(0.58–1.64)

OR.

Italics: statistically significant (P < 0.05).

Interactions between irrigation and livestock density on malaria prevalence.

a adjusted for socioeconomic & ecological characteristics, seasonality, altitude, use of mosquito nets, and residence.

**Fig. 3:**
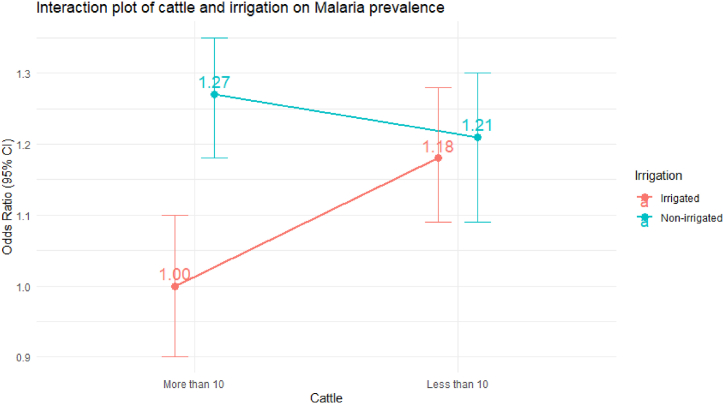
Interaction of livestock(cattle) and irrigation on malaria prevalence.

High density of cattle decreases malaria odds, but irrigation increases malaria odds. However high cattle number is not a significant protective factor in higher irrigation area. The interaction between cattle and irrigation has a significant impact on the malaria prevalence, but this impact depends on the level of irrigation (Fig. 3). In non-irrigated areas, low cattle presence is associated with higher odds of malaria prevalence compared to high cattle presence(P < 0.05). In irrigated areas, cattle presence does not significantly affect the odds of malaria prevalence (P < 0.812).

## 4 Discussion

Malaria is serious public health concern and a key barrier to achieving the sustainable development goals (SDGs)in Rwanda and sub-Saharan Africa in general. In this study examining malaria prevalence and its associated population and ecological risk factors in Rwanda, several significant findings that shed light on the complex dynamics of malaria in Rwanda was uncovered. The analysis revealed that several factors such as Non-use of ITN, insurance, education level, altitude, irrigation and living to the proximity of livestock like cattle were strongly associated with malaria prevalence, both positively and negatively.

### 4.1 Population-based factors

One of the striking observations was the significant association between malaria prevalence and education levels. Individuals with no formal education were found to be at a higher risk of malaria. This findings aligns with prior research, which has also noted a corresponding link between a level of education and the prevalence of malaria [18,19]. Individuals with lower levels of education may have limited health literacy and limited access to information about malaria prevention methods such as the use of insecticide-treated bed nets, indoor residual spraying therefore leading to an increased malaria transmission [20]. This finding underscores the importance of health education and awareness campaigns in vulnerable communities to promote the adoption of preventive measures such as ITN and proper malaria management.

The findings revealed a consistent association between malaria infection and the child's age, demonstrating that the likelihood of infection rises with the child's age. In previous studies, older children under the age of five exhibited a higher susceptibility to infection when compared with their younger counterparts [21,22]. Furthermore, older children face greater exposure to mosquitoes in comparison to younger children, as they are often less shielded by their parents. Another plausible explanation for the higher infection rate in older children is the fact that mosquitoes prefer biting older children rather than infants [23]. Therefore, Healthcare professionals should utilize various communication methods to effectively educate parents with older children under the age of five regarding the elevated malaria risk within this group. Timely information provided by healthcare workers can enhance parental awareness and knowledge about the heightened malaria risk for their children and promote preventive measures.

Non-use of ITN was another critical factor linked to increased malaria prevalence in Rwanda. Utilizing an ITN during sleep is the most commonly used preventive measure against malaria. The effectiveness of ITN lies in the fact that, in most malaria-endemic areas, the female mosquitoes responsible for transmitting malaria primarily bite during the night [24]. ITNs function as a protective barrier between individuals under the net and malaria causing mosquitoes. The chemicals in ITNs either repel or eliminate mosquitoes upon contact with the net. The use of ITN has shown prevent malaria cases compared to no net in various settlements [25]. The “community effect” is observed when a majority of people in a community use ITNs, leading to a collective reduction in the mosquito population and its lifespan. This, in turn, contributes to lowering the transmission of malaria [24]. Encouraging the consistent and proper use of ITNs, especially among those with lower educational backgrounds, is imperative to reduce malaria transmission.

The absence of health insurance was positively associated with malaria prevalence in this study. These results are in part influenced by the reduced health awareness and inequities in healthcare access. People without health insurance may be less likely to receive regular health check-ups or health education [26]. This reduced awareness can lead to a lack of knowledge about malaria prevention and early symptoms, further increasing the risk of infection [27,28]. Community-based health insurance (CBHI) is recognized as a viable route toward achieving universal health coverage in nations with low incomes [29]. CBHI provides financial protection [30,31] thus facilitating accessibility to health services [32,33]. As malaria disproportionately affects under-served populations including tribal people and migrant workers– the same populations who would benefit most from health insurance [34], enrolment in the CBHI program may increase the probability of utilizing a mosquito net and other preventive measures therefore reducing malaria incidence [24].

Higher wealth status was linked with a reduced malaria prevalence as found in previous studies [35,36]. This could be attributed to the fact that impoverished housing conditions often create environments conducive to mosquito breeding. A previous study conducted in Rwanda revealed that individuals residing in residences with inadequate wall materials and households affected by food shortages had a higher likelihood of contracting malaria [37]. Additionally, lower-income populations predominantly reside in rural areas where access to healthcare facilities, both physically and financially, is not as readily available as in urban areas. Furthermore, the expenses associated with medical consultations, transportation, and medications at healthcare facilities located far away can be a barrier for economically challenged families. Poverty can lead individuals to seek jobs agriculture as well as mining within forested regions, where malaria vectors are abundant [38,39]. Consequently, substandard housing and insufficient awareness about malaria contribute to the spread of the disease among impoverished communities. It's worth noting that malaria can perpetuate the cycle of poverty in developing world given that a substantial part of the population is dependent on cultivation, and the transmission of malaria aligns with farming and season of crops harvesting.

### 4.2 Ecological factors

Household members living at lower altitudes (<1700 m above sea level) were at a significantly higher risk of malaria (Fig. 2). Eastern and Southern regions of Rwanda possess the country's most minimal elevation levels which may explain their increased malaria prevalence compared to others. The variation in malaria incidence can be significantly attributed to geographical distinctions between lowland and highland regions [40].

Living in close proximity to irrigation areas was linked with malaria prevalence. This may highlight the complex interaction between irrigation practices and malaria transmission. A study conducted in western Ethiopia found a significant increased density of mosquitoes in proximity to the irrigation areas [41]. Irrigation can create new breeding sites for mosquito vectors of malaria. Irrigated fields, especially rice paddies, can provide stagnant water, which is ideal for mosquito larvae to develop. The availability of stagnant water due to irrigation can lead to higher populations of *Anopheles* mosquitoes in irrigated areas, increasing the risk of malaria transmission. To address the increased risk of malaria transmission associated with living near irrigation areas, an intervention focused on integrated vector management and community engagement is essential [42]. In irrigated schemes, using ITN that target susceptible vectors has proven to be an effective intervention for malaria control [42].

The finding that higher livestock (cattle) density is associated with a lower prevalence of malaria in Rwanda is intriguing and offers valuable insights into the complex dynamics of malaria transmission in Rwanda. This result aligns with some previous studies [11,12] but contradicts others that suggest that the existence of animals provides mosquitoes with a source of blood meals, ultimately extending their lifespan [13]; therefore emphasizing the need for careful consideration. Livestock, particularly cattle, can have a multifaceted influence on local ecosystems. Zoophilic tendency of malaria vectors in East African countries like Rwanda may explain the reduced prevalence of malaria but it can be influenced by other factors like distance between human dwellings and cattle, the use of ITN and other malaria control strategies [43]. Cattle may divert mosquitoes from humans to animals, thereby reducing malaria transmission [43,44]. In addition, cattle can create disturbances in vegetation and soil, which may inadvertently disrupt mosquito breeding sites. The trampling effect of cattle on potential mosquito larval habitats, such as small puddles or ponds, could reduce the number of available breeding sites. The presence of cattle might introduce competition for resources in these aquatic ecosystems, making it less favorable for mosquito larvae survival.

This study results hold significant clinical implications for malaria management and prevention strategies in Rwanda. Healthcare providers should prioritize malaria screening and treatment for vulnerable groups, particularly children aged 4 and above and individuals residing at lower elevations. Furthermore, targeted interventions focusing on environmental factors such as irrigation and livestock density may complement existing malaria control efforts, particularly in high-risk areas.

### 4.3 Study limitations and strengths

The cross-sectional design of the 2019–2020 Rwanda DHS makes it impossible to identify a cause and effect relationship. This limitation prevents making definitive conclusions regarding any association directions. Furthermore, testing for malaria was exclusively conducted in women and children under the age of 5, rather than the overall community, which restricts the generalizability of the findings to the entire population. In addition, while the Rwanda DHS collects a wide range of demographic and health-related information, certain variables relevant to malaria transmission like types of vectors, spraying, mosquito biting data, are not included in the survey. This limitation restricts the comprehensiveness of analyses and may overlook important determinants of malaria prevalence. While acknowledging these limitations, this study's results offer valuable insights into the existing population and environmental determinants of malaria in Rwanda. This study's findings provide insight on the influences of irrigation, livestock, insurance, and elevation on malaria prevalence. These findings can serve as a foundational resource for forthcoming research and can underpin the development of policies and initiatives dedicated to malaria eradication.

## 5 Conclusions

Limited studies were conducted to investigate the effects of irrigation and domestic animals like cattle on malaria within the general population. The findings validate that living at the proximity of irrigation areas has a substantial effect on the transmission of malaria. Non-use of mosquito nets, education level as well as income level also showed a significant association with malaria. This reiterates the significance of considering these elements when creating strategies and actions for malaria eradication. In addition, high cattle density has shown to have a protective effect on malaria transmission in Rwanda. To assess the association between season, residence type, and malaria prevalence more thoroughly, it's essential to conduct longitudinal studies, as these factors did not exhibit significance in this study. The identification of the interaction between livestock and irrigation in relation to malaria prevalence presents a significant opportunity to inform targeted policy recommendations for Rwanda. Given the role of livestock and irrigation practices in shaping local ecosystems and mosquito breeding habitats, policymakers can leverage these findings to implement integrated vector control strategies. Additionally, promoting sustainable irrigation practices can mitigate the creation of stagnant water bodies ideal for mosquito breeding. Implementing future measures to alleviate financial obstacles to preventive actions and reduce malaria susceptibility will result in decreased malaria prevalence. There's a crucial need for the effective utilization of ITN, necessitating an increased emphasis on community-wide ITN utilization. Moreover, when designing strategies for malaria control or eradication, regions at lower altitudes should be taken into account. Highlighting future research directions, comprehensive studies should consider the interactions between ecological factors to gain a holistic understanding of malaria transmission and develop more effective and sustainable interventions to combat this public health threat.

## Accessibility of data and resources

Public access to DHS datasets as well as questionnaire can be freely found at www.dhsprogram.org.

## Informed consent

Prior to conducting the DHS, IRB approval was secured from the inner city fund. Furthermore, all DHS study participants were fully informed about every aspect of the survey they would be involved in. Participants were also assured of the confidentiality of their information and the results of any clinical tests. Are assured of their confidentiality as well as their answers or results in case of clinical test.

## Funding

This work was supported by the National Research Foundation of Korea (BK21 Center for Integrative Response to Health Disasters, Graduate School of Public Health, Seoul National University)(NO.419 999 0514025).

## CRediT authorship contribution statement

**Guillaume Rudasingwa:** Writing – review & editing, Writing – original draft, Visualization, Software, Methodology, Formal analysis, Data curation, Conceptualization. **Sung-il Cho:** Writing – review & editing, Validation, Supervision, Investigation, Conceptualization.

## Declaration of competing interest

The authors declare that they have no known competing financial interests or personal relationships that could have appeared to influence the work reported in this paper.
